# Patterns of Comorbidities in Lung Cancer Patients and Survival

**DOI:** 10.3390/cancers17091577

**Published:** 2025-05-06

**Authors:** Alessandra Buja, Marcello Di Pumpo, Massimo Rugge, Manuel Zorzi, Federico Rea, Ilaria Pantaleo, Giovanna Scroccaro, Pierfranco Conte, Leonardo Rigon, Giorgio Arcara, Giulia Pasello, Valentina Guarneri

**Affiliations:** 1 Department of Cardiologic, Vascular and Thoracic Sciences, and Public Health, University of Padova, 35128 Padova, Italy; 2 Department of Life Sciences and Public Health, Università Cattolica del Sacro Cuore, 00168 Rome, Italy; marcello.dipumpo@unicatt.it; 3 Department of Prevention, AULSS6 Euganea, 35100 Padova, Italy; 4 Department of Medicine DIMED—Pathology Unit University of Padova, 35100 Padova, Italy; 5 Veneto Tumor Registry (RTV), Azienda Zero, Passaggio Luigi Gaudenzio, Padova 1, 35131 Padova, Italy; manuel.zorzi@azero.veneto.it; 6 Coordinamento Regionale per le Attività Oncologiche (CRAO), Regione Veneto, 30100 Venezia, Italy; 7 Istituto di Ricovero e Cura a Carattere Scientifico, San Camillo Hospital, Via Alberoni, Lido, Venezia 70, 30126 Venice, Italy; 8 Department of Neuroscience, University of Padova, 35127 Padova, Italy; 9 Department of General Psychology, University of Padova, 35127 Padova, Italy; 10 Oncologia Medica 2, Istituto Oncologico Veneto, Istituto di Ricovero e Cura a Carattere Scientifico, 35121 Padova, Italyvalentina.guarneri@unipd.it (V.G.); 11 Department of Surgery, Oncology and Gastroenterology, University of Padova, 35127 Padova, Italy

**Keywords:** comorbidity, survival, lung cancer, epidemiology, prognosis

## Abstract

Introduction: Comorbidities affect diagnosis and treatments in cancer patients. This study explores the prevalence and patterns of comorbidities in non-small cell lung cancer (NSCLC) patients and their association with survival. Materials and Methods: This retrospective population-based cohort study included 1674 incident NSCLC patients. Comorbidities were classified based on the ICD-9-CM system, with 13 disease categories analyzed. Patients with more than two comorbidities were classified into three mutually exclusive and exhaustive latent classes (Latent Class Analysis [LCA]). The optimal number of latent classes was determined by applying the Akaike Information Criterion. Cox regression models were run to assess overall and cancer-specific mortality, adjusting for the comorbidity groups, sex, age, and stage at diagnosis. Results: In 1674 NSCLC patients, the most prevalent medical conditions were respiratory (35.8%) and cardiovascular (33.5%). The Cox regression showed that even one comorbidity is associated with an increased hazard of overall mortality (HR = 1.33, 95%CI: 1.11–1.59, *p* = 0.002). LCA-derived Class-1 (cardiovascular-respiratory and endocrine) reported HR = 1.74 (95%CI: 1.39–2.17, *p* < 0.001), Class-2 (multi-organ) HR = 1.44 (95%CI: 1.18–1.77, *p* < 0.001), and Class-3 (socio-multifactorial-neuro) HR = 1.62 (95%CI: 1.36–1.93, *p* < 0.001). Instead, in patients with one comorbidity, NSCLC-specific mortality showed no significant trend towards increased risk (HR = 1.17, 95%CI: 1.00–1.43, *p* = 0.114). Significant associations emerged between NSCLC-specific mortality and LCA-classes: Class-1: HR = 1.49 (95%CI: 1.20–1.91, *p* = 0.001); Class-2 HR = 1.25 (95%CI: 1.0–1.57 *p* = 0.048); and Class-3: HR = 1.23 (95%CI: 1.00–1.48, *p* = 0.035). Conclusions: The adverse impact of comorbidities on NSCLC-specific mortality requires their inclusion as risk factors in cancer treatment and prognosis.

## Simple Summary

This study investigates the prevalence of comorbidities in a non-small cell lung cancer (NSCLC) cohort and evaluates how comorbidities impact overall and cancer-specific mortality in patients. By examining a population-based cohort of 1674 NSCLC cases, distinct comorbidity patterns were identified. The results showed that even a single comorbidity significantly increased overall mortality risk. In contrast, only specific comorbidity patterns adversely affected cancer-specific survival. The findings underscore the importance of considering a patient’s comorbidity profile when determining prognosis and treatment plans for lung cancer. This research highlights the need for more personalized, comorbidity-oriented approaches in oncology care. Integrating comorbidity assessments could refine risk stratification, guide therapeutic decisions, and ultimately improve outcomes for NSCLC patients with complex health conditions.

## 1. Introduction

In 2022, lung cancer was the most incident malignancy worldwide, with 2.48 million new cases reported [1,2]. Despite significant advancements in primary and secondary prevention and treatment strategies, lung cancer remains the leading cause of cancer-related mortality, accounting for nearly 1 in 5 cancer deaths globally and accounting for 1.82 million deaths in 2022 [1,3]. A major factor contributing to the poor prognosis is the high prevalence of comorbidities, defined as the coexistence of one or more additional diseases or medical conditions, in lung cancer patients.

Comorbidities, in fact, in cancer patients may increase the complexity of diagnostic and therapeutic procedures, limit surgical options and the patient’s enrolment in therapeutic trials, hinder the completion of chemotherapy regimens, and prevent the patient from receiving full-dose therapies; all these potential therapeutic restrictions ultimately impact cancer prognosis [4,5]. Beyond their direct effects on treatment delivery, comorbidities can also indirectly influence lung cancer prognosis through various mechanisms. For instance, the presence of chronic inflammatory states or metabolic dysregulation associated with certain comorbidities may promote a tumor-promoting microenvironment and accelerate cancer progression [4]. Conversely, some comorbidities and their treatments may confer protective effects against lung cancer through poorly understood biological mechanisms, as exemplified by the “obesity paradox” observed in certain studies [6].

Up to 88% of lung cancer patients have at least one additional medical condition, with a median of two comorbidities per individual [7] and previous studies found that comorbidities significantly affect prognosis, leading to a 21% increase in mortality rates within five years [8,9,10]. A study found that the number of comorbidities is an independent predictor of survival in elderly patients undergoing systemic chemotherapy, including EGFR-TKIs for advanced NSCLC [11]. The most prevalent medical conditions are hypertension and chronic obstructive pulmonary disease (COPD), consistently reported as affecting 28.4% to 39.8%, followed by metabolic and renal diseases [12,13,14]. A study on the effect of different comorbidities on the survival of non-small cell lung cancer patients found that patients with cardiovascular comorbidity have a 30% higher death rate than patients without comorbidity. Patients with diabetes and patients with cerebrovascular disorders and COPD have a 20% excess mortality compared to patients without comorbidity [15].

A comprehensive assessment of comorbid conditions can inform treatment strategies, improve prognostic estimates, and enhance the quality of life for patients. This study explores the prevalence of comorbidity patterns in NSCLC patients and examines their association with survival, addressing their implications from a public health standpoint.

## 2. Patients and Methods

### 2.1. Socio-Economic and Healthcare Setting

The Italian National Healthcare System is publicly funded through general taxation and administered at the regional level. Its core principles include universal access, fairness, and unrestricted choice of providers. Regional authorities oversee healthcare management, ensuring equitable care delivery throughout their territory. In 2013, the Veneto Regional Government established an interdisciplinary Oncology Network (ROV) dedicated to developing, implementing, and monitoring diagnostic and therapeutic pathways for oncology patients. Drawing from the best national and international clinical evidence, the ROV issues documents outlining criteria for cancer diagnostic procedures, treatment approaches, and end-of-life care across common cancer types. Notably, in 2022, the ROV released comprehensive guidelines detailing the diagnostic and therapeutic pathways (Italian acronym: PDTA) specifically for lung cancer patients, covering all stages from initial diagnosis through end-of-life support.

### 2.2. Study Population

This retrospective cohort study utilized population-based data from the Veneto Region in Northeast Italy, where comprehensive cancer recording covers all resident populations. The study analyzed a total of 1674 incident cases of non-small cell lung cancer (NSCLC) diagnosed across two years: in 2017 (850 cases) and 2019 (824 cases) across two Local Health Authorities (LHAs) in the Veneto Region of Northeastern Italy. In both LHAs, cancer recording involved the entire resident population. The clinical and pathological details of the NSCLC cases were obtained from the high-resolution Veneto Tumor Registry (Registro Tumori del Veneto [RTV]).

### 2.3. Comorbidities

The information on the comorbidity classes was obtained from the hospital records reporting the primary and secondary diagnoses classified according to the ICD9-CM system, as applied at the time of cancer incidence. Thirteen primary disease categories were analyzed (the presence/absence of major ICD9-CM disease categories and V codes). Only diagnoses within 6 months from hospitalization were considered [16,17].

Comorbidity groups were categorized as follows: (a) 0 = no comorbidities; (b) 1 = one comorbidity; and (c) Classes 1, 2, and 3 of comorbidity as resulting from Latent Class Analysis.

### 2.4. Statistical Analysis

Descriptive analyses profiled the costs and survival characteristics of NSCLC. Comorbidity patterns were identified using Latent Class Analysis (LCA). Patients with more than two concurrent diseases were allocated in three latent classes. The Akaike Information Criterion (AIC) determined the optimal number of mutually exclusive and exhaustive latent classes: the values of AIC were used in comparing several possible models, with the lowest value suggesting the best-fitting model.

Consequently, we assigned it a corresponding name by identifying the most characteristic comorbidities. The resulting latent class variable represents groups of like individuals and distinguishes them from other individuals who are less alike. The model estimates the posterior probability of class membership for each individual and subsequently places the individual in the class for which the probability of membership is highest. Kaplan–Meier curves were created to compare survival patterns at different comorbidity patterns. Cox regression models were run to study overall and NSCLC-specific mortality by the comorbidity groups, adjusting for sex, age, and stage at diagnosis.

The statistical packages R 3.6.2 and SAS 9.4 were used for record linkage and all statistical analyses.

### 2.5. Ethics

The analyses were conducted using anonymized, aggregated data that precluded any possibility of individual patient identification. Ethical approval for this study was granted by the ethics committee of the Veneto Oncological Institute (approval no. 03/2021).

## 3. Results

The study cohort included 1674 NSCLC patients with a mean of comorbidity of 2.09 ± 1.70. Among them, 951 patients (56.23%) had at least two comorbidities at the time of cancer diagnosis. The most prevalent medical conditions were respiratory (35.8%) and cardiovascular diseases (33.5%). Table 1 shows the general profile of the patients considered.

Eight hundred sixty-six patients with two or more comorbidities were allotted in the three identified LCA classes (Table 2). In particular, in Class 1 (*Cardiovascular-Respiratory and Endocrine*), dominant conditions included circulatory system diseases (99.91%) and respiratory diseases (83.16%) but also endocrine (32.54%); in Class 2 (*Multi-Organ:* Genito-Infectious-Hematologic-Digestive), they included patients with a higher prevalence of infectious diseases (26.62%), blood disorders (25.22%), digestive diseases (36.11%), and genitourinary diseases (35.31%); in Class 3 (*Socio-Multifactorial-Neuro Conditions*), the dominant conditions were factors influencing health status (59.27%) and symptoms or undefined conditions (36.93%), and neurological and sense organs disorders (16.71%).

**Figure 1:**
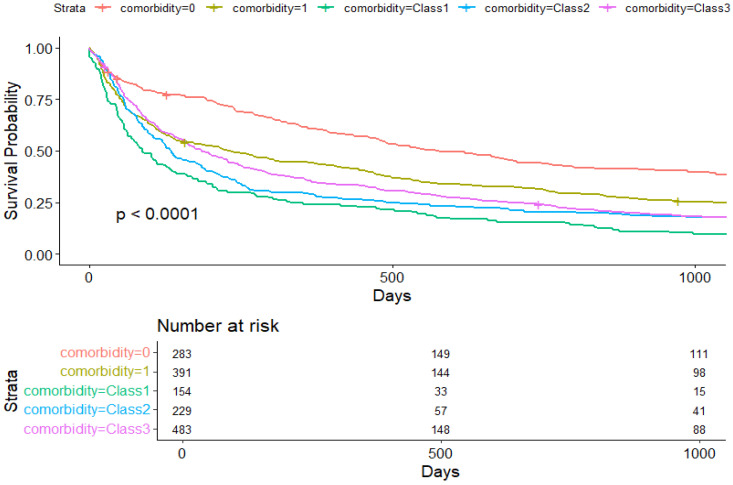
Overall causes of mortality by comorbidity groups.

**Figure 2:**
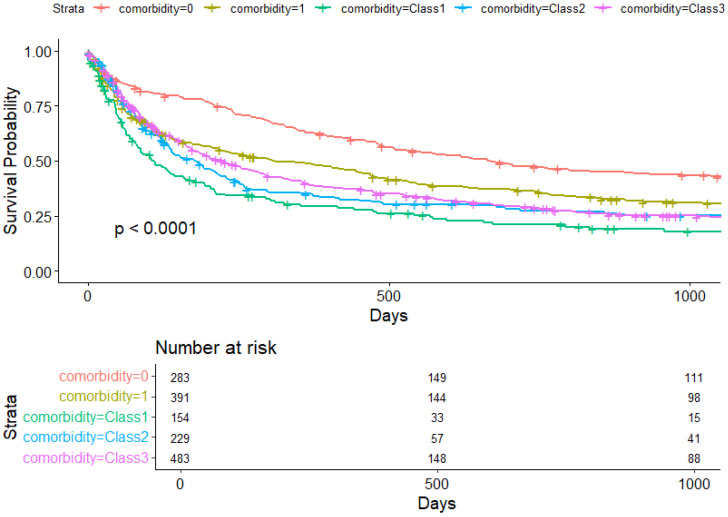
Lung cancer-specific mortality by comorbidity groups.

Figure 1 and Figure 2 show Kaplan–Meier overall and lung cancer-specific survival slopes by comorbidity groups, respectively.

The Cox regression model for overall survival included 1540 patients with 1263 deaths. The presence of one comorbidity was associated with an increased hazard of death (HR = 1.33, 95%CI: 1.11–1.59, *p* = 0.002). Hazard ratio values by LCA-derived classes were as follows: Class 1: HR = 1.74 (95% CI: 1.39–2.17, *p* < 0.001): Class 2: HR = 1.44 (95%CI: 1.18–1.77, *p* < 0.001); and Class 3: HR = 1.62 (95%CI: 1.36–1.93, *p* < 0.001) (Table 3).

In patients with a single comorbidity, NSCLC-specific mortality showed no significant trend towards increased risk (HR = 1.17, 95%CI: 1.00–1.43, *p* = 0.114). Significant associations emerged between NSCLC-specific mortality and LCA Class 1: HR = 1.49 (95%CI: 1.20–1.91, *p* = 0.001); LCA Class 2: HR = 1.25 (95%CI: 1.0–1.57, *p* = 0.048); and LCA Class 3: HR = 1.23 (95%CI: 1.00–1.48, *p* = 0.035).

## 4. Discussion

This retrospective population-based cohort study focuses on the impact of comorbidities on the prognosis of NSCLC patients.

Previously in the literature, in 2010, a review article on “comorbidity-oriented oncology” critically reconsidered the “traditional” use of age as a comprehensive indicator of a patient’s comorbidity status and its impact on cancer prognosis. While acknowledging age’s prognostic impact, the authors highlighted the importance of detailing additional clinical factors such as multiorgan functional profile, nutritional status, cognitive function, psychological well-being, social support, and the patient’s medications [18]. More than fifteen years later, Leduc and colleagues highlighted the importance of comorbidities in diagnosing and managing lung cancer patients. Based on epidemiological profiles and the natural history of cancer, the authors concluded that a significant proportion of patients with NSCLC represent a “special population” that requires diagnostic and therapeutic approaches tailored to their comorbidities [9,19]. Rogers and co-authors quantified the impact of comorbidity on the lung cancer diagnostic pathway. They documented that two or more comorbidities significantly prolonged the diagnostic workup from 31 to 74 days [20]. In a study on the medical conditions associated with bronchogenic carcinoma, Gould and co-authors analyzed the prognostic impact of comorbidities in 6662 NSCLC patients [21]. The latent class analysis identified five distinct comorbidity classes, each with an increasing comorbidity index: (a) Class 1 (60% of patients) showed a low comorbidity burden, mainly with mild chronic lung disease and diabetes; (b) Class 2 (17% of patients) showed a high prevalence of chronic obstructive pulmonary disease (COPD), heart failure, and peripheral vascular disease; (c) Class 3 (6% of patients) was characterized by cerebrovascular disease and respiratory and cardiac conditions; (d) Class 4 (12% of patients) included individuals with diabetes and severe complications such as renal disease; and (e) Class 5 (6% of patients) consisted of patients with severe multi-organ comorbidities. In patients with stage 0-II lung cancer, a significant correlation emerged between comorbidity class and both treatment selection and survival outcomes [22].

The present study identified three distinct comorbidity patterns: (a) Cardiovascular-Pulmonary and Endocrine Class; (b) Socio-Multifactorial-Neuro Class; and (c) Multi-Organ Disease Class, including a broad spectrum of multiorgan conditions involving infectious, hematologic, digestive, and genitourinary diseases. The clinical rationale for our findings is consistent with Leduc’s physio-pathological interpretation, which, in part, views the pattern of lung cancer comorbidities as a result of shared risk factors and interactions between diseases [19]. Examples include the coexistence of COPD and emphysema, both due to a history of smoking; ischaemic heart disease and hypertension, which are common in elderly patients and may complicate anticancer treatment (e.g., Class 1 of our results); and diabetes-related renal insufficiency with metabolic complications leading to low tolerance to anticancer therapies (e.g., Class 2 of our results). Severe COPD and emphysema are associated with worse survival outcomes after surgery, particularly in men and in those with squamous-cell carcinomas. As documented by Leduc and coauthors, cardiovascular diseases (CVDs) affect 23% of lung cancer patients, lower overall survival, and increase the risk of non-cancer-related deaths. Interstitial lung diseases (ILDs) significantly increase postoperative mortality and contribute to poor long-term survival, especially in early-stage NSCLC [19].

Based on German health insurance data, Murawski and coauthors analyzed the five-year prognostic baseline impact of “comorbidome” in a cohort of 16,202 lung cancer patients. Comorbidities (e.g., paralysis, heart failure, and electrolyte disorders) are linked to higher mortality. However, as also reported in other studies, Murawski and coauthors demonstrated an inverse correlation between survival and weight loss at presentation, and a protective effect of obesity (so-called “obesity paradox”) was associated with more prolonged survival [6].

The present study provides evidence that the presence of any comorbidity significantly increases the overall risk of mortality in this population after adjusting for stage at diagnosis. All patterns of comorbidities examined in the study showed a significant impact on cancer-specific mortality rates, indicating that any comorbidity pattern, regardless of its specific nature, negatively affects cancer prognosis. These findings align with those reported by Gould and colleagues, who discovered that NSCLC patients with diabetes, cardiovascular diseases, and chronic lung conditions had a lower likelihood of survival than those with fewer comorbidities [23]. An explanation could be that the concurrences of medical conditions affected the therapeutic choice or the competition of the treatment’s protocols, ultimately affecting cancer survival. Moreover, patients with multiple comorbidities tended to receive less aggressive/effective treatments, potentially reducing their survival chances. Concerning treatment implications, comorbidities significantly influence treatment choices, often limiting the feasibility of appropriate therapies [23,24].

The results of this study, along with similar research, strongly suggest that the diagnostic and treatment pathways for lung cancer need to be reconsidered in a broader clinical context. Based on these results, treatment strategies should be tailored to the overall health status of each patient. While standardized protocols have led to significant advances in diagnosis and treatment, they often overlook the unique clinical factors of each patient, as well as the individual clinical factors that are unique to each patient. This approach enhances clinical performance, patient satisfaction, and the effective allocation of resources by preventing the fragmentation of care [25,26].

The retrospective design of this study results in its non-trivial limitations; among them, respiratory comorbidities can indeed be difficult to separate from respiratory alterations induced by the tumor itself. However, no data were available in our sample to perform this distinction, using only ICD9-CM, recorded in dischargee records. Moreover, significant limits are represented by the lack of integration in the analysis of the treatment regimens and the patients’ adherence to the diagnostic therapeutic protocols. In particular, the lack of information on treatment patterns, including the type, intensity, and timing of systemic therapies, surgery, or radiotherapy, represents a critical limitation in determining whether the comorbidities pattern directly affects survival or indirectly influences treatment management. These potential weaknesses are partially balanced by the present population-based cohort study design. In fact, the population-based sample records all incident cases in the two LHAs in the Veneto Region. Data do not have a hospital-based or center-specific approach, which could undermine the generalizability of the results to the entire incidence case population. Moreover, the concordant results with previous literature could suggest the external validity of our results [21]. However, future prospective studies should validate these findings and explore corrective interventions tailored to specific comorbidity profiles of NSLC patients [27].

## 5. Conclusions

In conclusion, comorbidities impact the prognosis of NSCLC patients. Given the substantial impact of comorbidity on lung cancer management, future research should focus on refining the ranking of the patient’s risk stratification, integrating comorbidity burden into treatment decision-making. These reshaped models are critical to personalizing the opportunities offered by precision medicine and selecting therapies targeted at patients’ comorbidome.

## Figures and Tables

**Table 1:** NSCLC population by gender and age class.

	Number (%)	Mean (%) Comorbidities
GENDER		
Females	611 (36.50)	1.93 (51.81)
Males	1063 (63.50)	2,18 (58.72)
AGE CLASS AT DIAGNOSIS		
<65	316 (18.88)	1.89 (49.83)
65–69	207 (12.37)	1.99 (54.74)
70–74	328 (19.59)	1.90 (52.61)
75–79	3509 (18.46)	2.11 (56.94)
80–84	245 (14.64)	2.28 (62.66)
>85	269 (16.07)	2.44 (62.55)
NUMBER OF COMORBIDITIES		
0	283 (16.91)	
1	391 (23.36)	
2	309 (18.46)	
3	245 (14.64)	
4	172 (10.27)	
5	82 (4.9)	
6	37 (2.21)	
7	16 (0.96)	
≥8	5 (0.30)	
Missing data	134 (8.0)	
ICD9-CM DISEASE CATEGORY CLASSIFICATION		
Respiratory System	608 (36.32)	
Circulatory System	596 (35.6)	
Factors Influencing Health Status (V_codes)	404 (24.13)	
Symptoms, Signs, and Undefined Conditions	291 (17.38)	
Endocrine, Nutrition, Metabolism, and Immune Disorders	231 (13.8)	
Genitourinary System	178 (10.63)	
Blood and Hematopoietic Organs	164 (9.8)	
Digestive System	163 (9.74)	
Injuries and Poisoning	151 (9.02)	
Nervous System and Sense Organs	123 (7.35)	
Musculoskeletal System and Connective Tissue	117 (6.99)	
Infectious and Parasitic Diseases	89 (5.32)	
Mental Disorders	69 (4.12)	

**Table 2:** Probability (%) that a patient belongs to latent Classes 1, 2, and 3 for each disease group. Class 1 includes cardiovascular-respiratory and endocrine diseases; Class 2 includes multiorgan diseases; and Class 3 includes socio-multifactorial neuro conditions.

ICD9-CM Disease Category Classification	Class 1	Class 2	Class 3
Infectious and Parasitic Diseases	0.01%	26.62%	2.35%
Endocrine, Nutrition, Metabolism, and Immune Disorders	32.54%	26.91%	20.79%
Blood and Hematopoietic Organs	8.78%	25.22%	15.76%
Mental Disorders	7.16%	6.11%	8.26%
Nervous System and Sense Organs	6.86%	6.61%	16.71%
Circulatory System	99.91%	52.57%	51.44%
Respiratory System	83.16%	45.34%	56.93%
Digestive System	3.44%	36.11%	9.58%
Genitourinary System	19.12%	35.31%	10.13%
Musculoskeletal and Connective Tissue	4.53%	15.08%	12.51%
Symptoms, Signs, and Undefined Conditions	10.48%	25.74%	36.93%
Injuries and Poisoning	1.08%	19.99%	17.32%
Factors Influencing Health Status (V codes)	1.90%	29.58%	59.27%

**Table 3:** Cox survival analysis adjusting for comorbidity groups, gender, age, and NSCLC stage.

	All-Cause Deaths		Lung Cancer Deaths	
	HR (Lower-Upper)	*p*-Value	HR (Lower-Upper)	*p*-Value
Presence of comorbidity	1.33 (1.11–1.59)	0.002	1.17 (1.0–1.43)	0.114
Comorbidity Class 1	1.74 (1.39–2.17)	<0.001	1.49 (1.2–1.91)	0.001
Comorbidity Class 2	1.44 (1.18–1.77)	<0.001	1.25 (1.0–1.57)	0.048
Comorbidity Class 3	1.62 (1.36–1.93)	<0.001	1.23 (1.0–1.48)	0.035
Male gender	1.26 (1.12–1.42)	<0.001	1.11 (1.0–1.26)	0.130
Age	1.03 (1.03–1.04)	<0.001	1.02 (1.0–1.03)	0.000
Stage II	2.54 (1.64–3.94)	<0.001	1.61 (0.8–3.21)	0.177
Stage III	5.27 (3.70–7.51)	<0.001	3.12 (1.7–5.67)	<0.001
Stage IV	11.95 (8.59–16.63)	<0.001	5.32 (3.0–9.45)	<0.001
Missing stage	11.01 (6.97–17.39)	<0.001	9.17 (4.7–17.94)	<0.001

## Data Availability

The data supporting the findings of this study are held by the Veneto Epidemiological Registry and were used under license for the present work, but they are not publicly available. These data are nonetheless available from Manuel Zorzi on reasonable request and subject to permission to be obtained from the Veneto Epidemiological Registry (Veneto Regional Authority).
